# An artificial intelligence-based diagnosis system for the identification of helminth parasitic infections in mithun and allied bovines

**DOI:** 10.1038/s41598-025-32775-4

**Published:** 2025-12-17

**Authors:** Jayanta Kumar Chamuah, Bikash Sarma, Angughali Aheto Sumi, Mahak Singh, Harshit Kumar, J. Arul Valan, S. Girish Patil

**Affiliations:** 1 ICAR-NRC on Mithun, Medziphema, Nagaland, 797106 India; 2 National Institute of Technology, Chümoukedima, Nagaland, 797103 India; 3 ICAR-Research Complex for NEH Region, Medziphema, Nagaland 797106 India

**Keywords:** Computer vision, Parasitic identification, Livestock health, Convolutional neural networks, Veterinary diagnostics, Machine learning, Zoology

## Abstract

This study presents a novel deep learning approach addressing the critical shortage of veterinary expertise in India’s North Eastern Hill (NEH) region through automated identification of parasitic infections in livestock. We developed a Convolutional Neural Network (CNN) architecture capable of analyzing both standard and microscopic images to identify and classify 16 distinct parasitic species. The model comprises four convolutional layers (32, 64, 128, 256 filters) with ReLU activation and MaxPooling for efficient feature extraction, followed by Dense layers and a Softmax classifier. The model was trained on a comprehensive dataset of over 5,334 annotated images, achieving 96% accuracy after 30 training epochs. To evaluate stability, it was trained ten times, yielding an average accuracy of 0.9616 ± 0.0024 (95% CI: [0.9601, 0.9630]), Macro F1 of 0.9527 ± 0.0021, and Weighted F1 of 0.9598 ± 0.0019, demonstrating consistent performance. A PHP-based web interface enables real-time predictions and adaptable deployment across hardware and cloud platforms. This system offers a scalable and accessible diagnostic tool for enhancing parasite detection and livestock health monitoring.

## Introduction

Parasitic infections pose significant challenges to livestock health and agricultural productivity worldwide, often manifesting through subclinical symptoms that result in reduced growth rates, reduced milk production and carcass quality, and reduced fertility^1–3^. While microscopy remains the gold standard for parasitic diagnosis in livestock, this method presents considerable limitations: it is time-consuming, labor-intensive, and requires both specialized equipment and highly trained personnel. These diagnostic challenges are particularly acute in India’s North Eastern Hill (NEH) region, where geographical isolation and limited resources create significant barriers to accessing veterinary expertise. The resulting delays in parasite identification and treatment lead to substantial economic losses for farmers through decreased milk production, reduced meat yield, and in severe cases, livestock mortality. This regional crisis necessitates an innovative approach to parasitic diagnosis that can overcome both geographical and resource constraints. Artificial Intelligence (AI), particularly computer vision technology, offers a promising solution to this challenge. The effectiveness of traditional image understanding is limited by the scarcity of human experts, expert fatigue, high consultation charges, and rough estimation procedures. Medical anomalies present additional challenges due to their highly variable shapes, locations, and structures^4^. While machine learning techniques can help overcome these limitations, they traditionally require manual extraction of discriminant features. Convolutional Neural Networks (CNNs) represent a significant advancement by automatically learning and extracting necessary features for efficient image understanding^5,6^.

This study presents an AI-powered diagnostic system capable of identifying parasitic infections through both microscopic and normal images of helminth parasites. Our approach employs CNNs to develop a user-friendly model accessible via smartphones, making expert-level diagnostic capabilities available to farmers and veterinarians in remote areas. This approach aims to overcome issues such as the shortage of trained professionals in the NEH region, the time-intensive nature of traditional microscopic analysis, and the need for accessible diagnostic tools in geographically isolated areas.

## Materials and methods

Our proposed system is based on a Convolutional Neural Network (CNN) which is meticulously designed to detect and classify 16 distinct varieties of parasites from provided images. This model automates the identification process, offering a reliable tool for parasitologists and medical professionals to enhance diagnostic accuracy and efficiency. With a user-friendly interface, the system simplifies the task of parasite identification, making it accessible for both expert and non-expert users. The workflow for our parasite detection system involves several key stages: data preprocessing to enhance and prepare the image dataset, model training and validation to ensure accuracy and robustness, and the deployment of a user-friendly interface for real-time image classification. This comprehensive process ensures that the system is both effective and accessible, enabling users to quickly and accurately identify parasite species from uploaded images.

### Data pre-processing

We used TensorFlow’s Image Data Generator to perform data augmentation, a crucial step in improving our model’s performance and generalization ability. Data augmentation enhances the training dataset by applying different transformations to the original images, generating new and varied versions of each image. To achieve the final image dataset, we exercised the following operations which includes rescaling, rotation, width shifting, height shifting, sharing, zooming and flipping. Rescaling adjusts the pixel values of images to a normalized range of 0 to 1, which improves the model’s convergence during training by ensuring consistent input values. Rotation, on the other hand, randomly alters the images within a specified range (e.g., -20 to + 20 degrees) to simulate various orientations of the parasites. Width and Height Shifts randomly move images horizontally or vertically, ensuring the model can recognize parasites even when they appear off-centre in the image. Similarly shearing introduces a slight distortion by slanting the images, enabling the model to recognize parasites that may appear skewed or distorted in real-world scenarios. Additionally, random zooming in on images allows the model to learn to detect parasites at varying scales, improving its ability to accurately identify both large and small specimens. These augmentations enhance the model’s robustness and adaptability, ensuring it performs well under different conditions and variations in the input data. Flipping images horizontally introduces mirrored versions, allowing the model to identify parasites regardless of their left-right orientation. By applying these transformations, Image Data Generator creates a more diverse and extensive training dataset, which reduces the overfitting risk. Overfitting happens when a model excels on the training data but struggles to generalize to new, unseen data. Data augmentation mitigates this by exposing the model to a diverse range of image conditions, making it more robust and improving its performance on validation and test datasets.

### Model architecture

The proposed CNN is structured with multiple layers, each serving a specific function to extract and process features from the input images. The architecture is carefully crafted to balance complexity and computational efficiency, ensuring that the model can learn intricate patterns in the data while remaining scalable and fast. Each component, from the convolutional layers to the final dense layers, is chosen to maximize the model’s capability to generalize well across diverse parasite images, leading to high classification accuracy and robust performance.

**Fig. 1:**
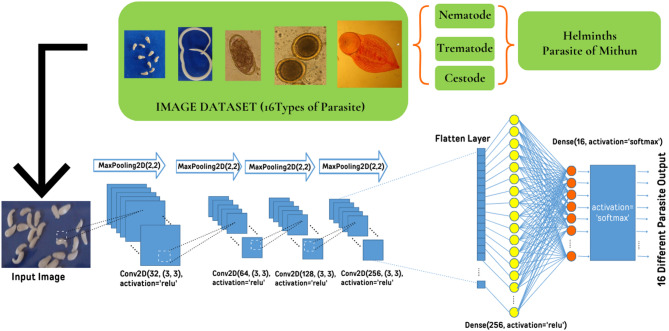
The CNN Model for identification of Helminth Parasite.

We chose the Sequential model for its straightforward approach to building neural networks by stacking layers in a linear order, where each layer’s output serves as the input to the next. This simplicity makes it easy to construct and understand the flow of data through the network, particularly in CNN architectures where layers like convolutional, pooling, and dense are typically arranged in a specific sequence. The Sequential model is ideal for our use case because it allows us to incrementally build a deep network layer by layer, ensuring that each layer progressively refines the features extracted from the input images.

Our model incorporates four convolutional layers, each with an increasing number of filters starting with 32 and increasing to 64, 128, and 256 to capture progressively more complex features from the input images which is represented in Fig. 1. The first layer starts with 32 filters to identify basic features such as edges and textures, while the subsequent layers increase the filter count, allowing the network to recognize more detailed and abstract patterns, such as shapes and structures unique to each parasite species. The choice of a 3 × 3 kernel size is deliberate; it is a standard size that is small enough to capture fine details in the images, such as the texture of a parasite’s surface, yet large enough to recognize more significant features when combined across multiple layers. By stacking these convolutional layers with increasing filter sizes, the model builds a hierarchy of features, from simple to complex, which is crucial for accurately distinguishing between the 16 different parasite classes in our dataset. The details of the proposed architecture are given in the Table 1.

**Table 1:** Architecture of the proposed custom CNN Model.

Layer No.	Layer Type	Filters/Units	Activation	Output Shape
1	Conv2D	32	ReLU	(None, 198, 198, 32)
2	MaxPooling2D	–	–	(None, 99, 99, 32)
3	Conv2D	64	ReLU	(None, 97, 97, 64)
4	MaxPooling2D	–	–	(None, 48, 48, 64)
5	Conv2D	128	ReLU	(None, 46, 46, 128)
6	MaxPooling2D	–	–	(None, 23, 23, 128)
7	Conv2D	256	ReLU	(None, 21, 21, 256)
8	MaxPooling2D	–	–	(None, 10, 10, 256)
9	Flatten	–	–	(None, 25600)
10	Dense	256	ReLU	(None, 256)
11	Dense	16	Softmax	(None, 16)

We selected ReLU (Rectified Linear Unit) as the activation function for our convolutional layers due to its ability to effectively introduce non-linearity into the model. Non-linearity is crucial because it enables the network to learn complex patterns and relationships that a linear model could not capture. ReLU works by outputting the input directly if it is positive and zero otherwise, which is simple yet powerful. ReLU is computationally efficient because it involves minimal calculations, allowing for faster training times compared to other activation functions like sigmoid or ReLU also helps address the vanishing gradient problem, a common challenge in deep networks where gradients become too small during backpropagation, potentially slowing or halting the learning process. By allowing gradients to pass through more effectively, ReLU ensures that the network can continue to learn and refine its feature extraction as the data moves through the layers, leading to better overall performance and faster convergence during training. After each convolutional layer, we incorporated MaxPooling layers with a 2 × 2 pool size to systematically decrease the spatial dimensions of the feature maps while holding the vital information. MaxPooling operates by sliding a 2 × 2 window across the feature map and selecting the maximum value within each window. This process not only reduces the size of the feature maps, which decreases the computational load and memory requirements but also helps in making the model more invariant to small translations and distortions in the input images. By reducing the spatial dimensions, MaxPooling layers effectively downsample the data, allowing the model to focus on the most dominant features detected by the convolutional layers. This dimensionality reduction also helps in preventing overfitting by making the model less sensitive to small variations in the training data. The 2 × 2 pool size is a standard choice that strikes a balance between reducing the dimensionality and retaining sufficient spatial information for accurate classification.

The flattened layer serves as a crucial bridge between the convolutional and fully connected (dense) layers in our CNN architecture. After the series of convolutional and pooling layers, the data is still in the form of multi-dimensional arrays (2D matrices) that represent the spatial features extracted from the input images. However, fully connected layers require input in the form of a one-dimensional (1D) vector. The Flatten layer performs this transformation by taking the 2D matrix of feature maps and converting it into a 1D vector, effectively flattening the data while preserving the spatial relationships learned by the previous layers. This step is necessary to pass the information from the convolutional layers to the dense layers, where higher-level reasoning and classification tasks are performed. By flattening the data, we allow the dense layers to process the extracted features holistically, combining all the learned patterns to make final predictions. This transformation enables the model to integrate the spatial information from the convolutional layers into a format suitable for classification, ensuring that the rich features captured by the CNN can be fully utilized in determining the class of the input image. Dense layers, also known as fully connected layers, play a vital role in integrating and interpreting the features extracted by the convolutional layers. In our model, we used two dense layers with specific purposes; First Dense Layer is responsible for learning complex, high-level features by combining the flattened features from the convolutional layers. It contains 256 neurons, each connected to every neuron in the previous layer, allowing it to capture intricate patterns and relationships. The ReLU activation function is used here to introduce non-linearity, enabling the model to learn more complex representations. It helps in activating the most relevant features while maintaining computational efficiency and avoiding issues like the vanishing gradient problem. The Second Dense Layer outputs the model’s prediction. It has 16 neurons, corresponding to the 16 parasite categories we aim to classify. Each neuron represents a potential class, and the softmax activation function is applied to convert the raw scores (logits) into probabilities. Softmax ensures that the output values sum to 1 and are interpretable as probabilities, indicating the likelihood of each class. This layer ultimately provides the final classification by selecting the class with the highest probability. Together, these dense layers facilitate the transition from feature extraction to classification, enabling the model to make accurate predictions based on the complex features learned from the images.

We chose the Adam optimizer for its advanced adaptive learning rate capabilities, which significantly enhance the training process of our CNN model. Adam, short for Adaptive Moment Estimation, combines the benefits of two other popular optimizers: AdaGrad and RMSprop. Unlike traditional optimizers such as Stochastic Gradient Descent (SGD), which use a fixed learning rate throughout training, Adam adjusts the learning rates for each parameter individually. It calculates adaptive learning rates based on estimates of the first and second moments (mean and uncentered variance) of the gradients. This means that parameters with larger gradients get a smaller learning rate, while those with smaller gradients get a larger one, allowing for more efficient and stable updates. Adam incorporates momentum-like terms to accumulate gradients over time. This helps in smoothing out the updates and accelerating convergence, especially in the presence of noisy gradients or sparse data. Adam includes mechanisms to correct biases in the estimates of the moments, especially during the early stages of training, ensuring more accurate parameter updates. Overall, Adam’s adaptive learning rate and momentum features make it highly effective in navigating complex loss landscapes, leading to faster convergence and better performance compared to traditional optimizers like SGD. This is particularly valuable in deep learning, where fine-tuning and optimizing model parameters can be challenging and computationally intensive. We selected Categorical Crossentropy as the loss function for our multi-class classification task because it is specifically designed to handle problems where each input belongs to one of several possible classes. This loss function evaluates the difference between the predicted probability distribution, generated by the model’s softmax output, and the true class labels, which are represented as one-hot encoded vectors. Categorical Crossentropy calculates the loss as the negative log of the predicted probability assigned to the actual class, penalizing incorrect predictions more heavily and guiding the model to improve its accuracy by adjusting its parameters to minimize this loss. This approach is particularly effective for training models in multi-class settings, ensuring that the predictions closely align with the true labels and enhancing overall classification performance.

**Fig. 2:**
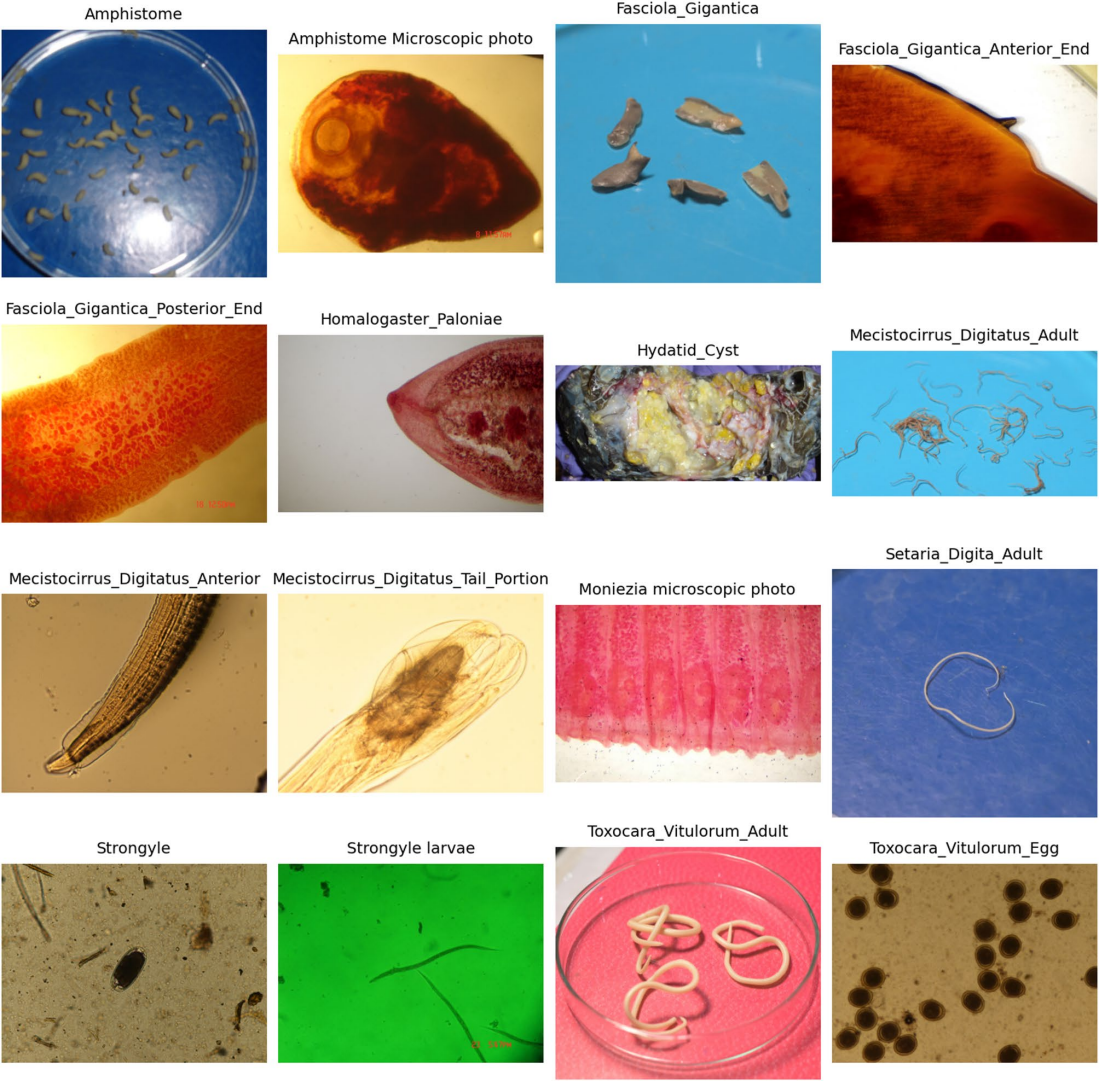
Training dataset provided to the system comprising 16 distinct types of images.

## Implementation

We developed the model using the TensorFlow framework, which offers a wide range of predefined libraries and functions that are both powerful and easy to implement. The experiments were conducted in Python with TensorFlow 2.10.0 and Keras 2.10.0 for model development and training. NumPy 1.24.4 was employed for numerical computations, while scikit-learn 1.4.1.post1 was used for evaluation metrics such as precision, recall, F1-score, and the confusion matrix. Matplotlib 3.7.2 and Seaborn 0.12.2 were utilized for visualizations. The different parasite classes considered in our proposed model are shown in Fig. 2. We used a dataset consisting of 5334 labeled images across 16 parasite classes. To ensure transparency and reproducibility, the class distribution is summarized in Table 2, which shows the exact number of images available in each class. All images in the dataset had resolutions ranging from 720 × 720 to 2048 × 2048 pixels and were saved in JPEG/PNG format. For the purpose of CNN training and testing, all images were resized to 200 × 200 pixels by setting img_height and img_width to 200. The normal images were captured using a 50-megapixel camera, while the microscopic images were obtained using a laboratory microscope (Dewinter, 99050554). The dataset was divided into three subsets: 70% for training, 15% for validation, and 15% for testing. The split was performed at the image level using scikit-learn’s train_test_split function with stratification to preserve class balance across subsets. A fixed random seed (random_state = 42) was applied to ensure reproducibility of results. The validation set allowed us to monitor the model’s performance on unseen data during training, providing an early indication of how well the model would generalize and helping to prevent overfitting. For evaluating our model, we used multiple metrics. Accuracy was chosen as the primary metric because it directly reflects the proportion of correctly classified images out of the total, while precision, recall, and F1-score were also reported to provide a more comprehensive assessment of the model’s ability to distinguish between the 16 parasite classes, especially in cases of class imbalance.

**Table 2:** Class distribution of 16 parasite Class.

Parasite Class	Number of Images			
	Train	Validation	Test	Total
Amphistome	437	94	94	625
Amphistome Microscopic photo	566	121	122	809
Fasciola_gigantica	129	28	28	185
Fasciola_gigantica_Anterior_End	373	80	81	534
Fasciola_gigantica_Posterior_End	265	57	57	379
Homalogaster_paloniae	237	51	52	340
Hydatid_Cyst	144	31	31	206
Mecistocirrus_digitatus_Adult	120	26	26	172
Mecistocirrus_digitatus_Anterior	192	41	42	275
Mecistocirrus_digitatus_Tail_Portion	259	56	56	371
Moniezia microscopic photo	265	57	57	379
Setaria_digita_Adult	242	52	52	346
Strongyle	104	22	23	149
Strongyle larvae	109	24	24	157
Toxocara_vitulorum_Adult	215	46	47	308
Toxocara_vitulorum_Egg	69	15	15	99
Dataset Size (Total Images)				5334

**Fig. 3:**
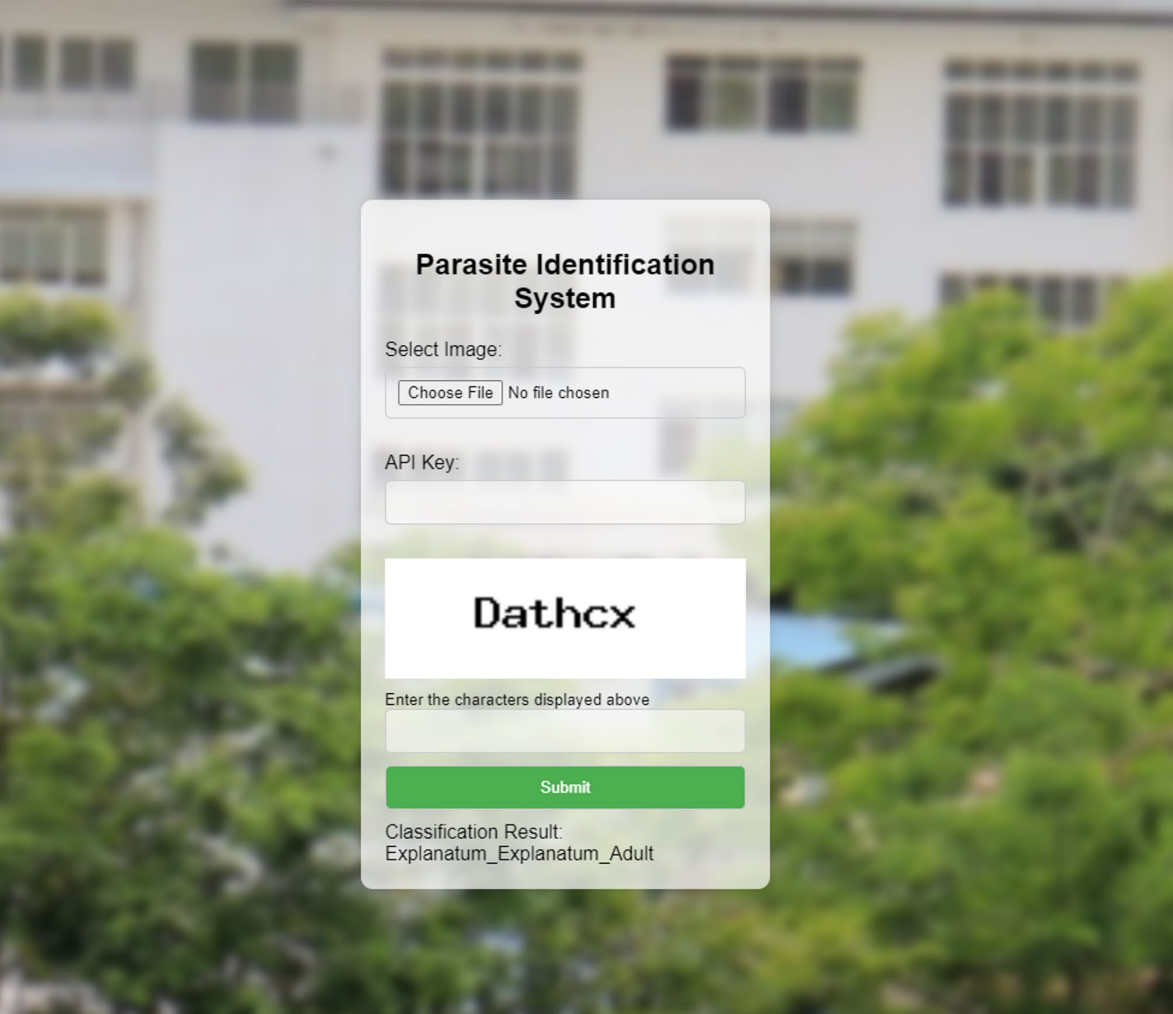
User Interface to upload the image and obtain the result in common human readable Format.

A Flask application was developed to serve the trained model and handle image classification requests, providing a user-friendly interface for real-time predictions. The API is secured with API key verification, ensuring that only authorized users can access the service. Before making predictions, images are pre-processed through a custom function that resizes and normalizes them to match the model’s input requirements, ensuring consistent and accurate results. The model’s output, which is a predicted probability distribution, is then mapped to human-readable class labels representing the different parasite categories, allowing users to easily understand the classification results. Figure 3 represents the developed interface, which allows image upload for parasite classification, secures access with API key protection, and displays the results.

## Results

The performance of the proposed custom CNN model was evaluated on the parasite image dataset consisting of 16 classes. The classification metrics, including precision, recall, and F1-score, were computed for each class to assess the effectiveness of the model. Table 3 presents the detailed results across all parasite classes. The model demonstrated high overall accuracy of 96.16%, with a macro-average F1-score of 95.14% and a weighted-average F1-score of 96.14%, indicating consistent performance across the dataset. Several classes such as Moniezia microscopic photo and Amphistome Microscopic photo achieved perfect or near-perfect classification, with F1-scores of 1.0 and 0.996, respectively. For Fasciola gigantica and its sub-classes (anterior and posterior ends), the model maintained robust classification, with F1-scores of 0.873, 0.988, and 0.983, respectively. Similarly, Mecistocirrus digitatus classes exhibited satisfactory results, with F1-scores ranging from 0.907 to 0.961, despite some intra-class variations due to morphological similarities. Classes with comparatively lower support, such as Strongyle and Toxocara vitulorum egg, also achieved reliable detection, with F1-scores above 0.93, reflecting the generalizability of the CNN model across different parasite types. Additionally, we evaluated the model performance using Top-3 and Top-5 accuracy metrics, which measure the proportion of cases where the correct class is within the top three and top five predicted probabilities, respectively. The model achieved a Top-3 accuracy of 99.13% and a Top-5 accuracy of 99.75%, demonstrating its strong ability to rank the correct class among the most likely predictions even when the top-1 prediction was incorrect.

**Table 3:** Precision, recall and F1 score of each class (Custom CNN).

Class Short Name	Parasite Class	Precision	Recall	f1-score	Support
C1	Amphistome	0.957895	0.968085	0.962963	94
C2	Amphistome Microscopic photo	0.99187	1	0.995918	122
C3	Fasciola_gigantica	0.888889	0.857143	0.872727	28
C4	Fasciola_gigantica_Anterior_End	1	0.975309	0.9875	81
C5	Fasciola_gigantica_Posterior_End	0.966102	1	0.982759	57
C6	Homalogaster_paloniae	1	0.980769	0.990291	52
C7	Hydatid_Cyst	0.96875	1	0.984127	31
C8	Mecistocirrus_digitatus_Adult	0.961538	0.961538	0.961538	26
C9	Mecistocirrus_digitatus_Anterior	0.886364	0.928571	0.906977	42
C10	Mecistocirrus_digitatus_Tail_Portion	0.943396	0.892857	0.917431	56
C11	Moniezia microscopic photo	1	1	1	57
C12	Setaria_digita_Adult	0.943396	0.961538	0.952381	52
C13	Strongyle	0.916667	0.956522	0.93617	23
C14	Strongyle larvae	0.956522	0.916667	0.93617	24
C15	Toxocara_vitulorum_Adult	0.913043	0.893617	0.903226	47
C16	Toxocara_vitulorum_Egg	0.933333	0.933333	0.933333	15
	Accuracy	0.961586	0.961586	0.961586	0.961586
	Macro Avg	0.951735	0.951622	0.95147	807
	Weighted Avg	0.961716	0.961586	0.961474	807

**Fig. 4:**
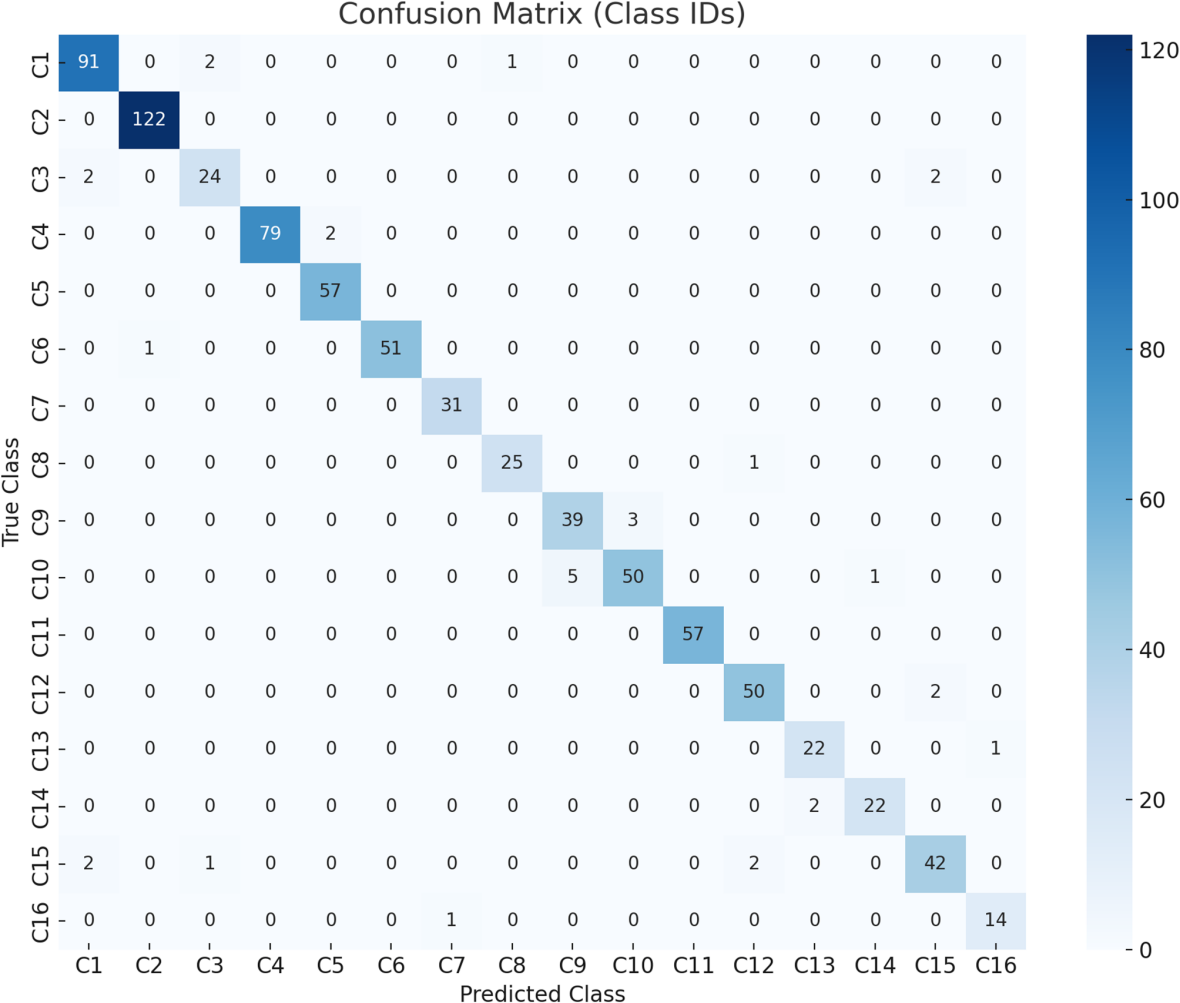
Heat map of the Confusion Matrix of Proposed CNN.

The confusion matrix in Fig. 4, provides deeper insight into the classification behavior of the proposed CNN model across the 16 parasite classes. The diagonal dominance of the matrix clearly indicates that the majority of samples are correctly classified into their respective categories, with only a few misclassifications observed. The Amphistome class achieved high recognition, with 91 out of 94 samples correctly classified, while minor confusion was observed with Fasciola gigantica (2 samples) and Mecistocirrus digitatus adult (1 sample). Amphistome microscopic photo achieved perfect classification (122/122), demonstrating the model’s strong ability to distinguish microscopic representations from macroscopic images. For Fasciola gigantica, 24 out of 28 samples were classified correctly, with a small overlap toward Amphistome and Toxocara vitulorum adult. Sub-class analysis revealed strong consistency: the anterior end achieved 79 correct predictions out of 81, and the posterior end achieved perfect classification (57/57). Similarly, Homalogaster paloniae recorded 51/52 correct predictions, with only one sample misclassified as Amphistome microscopic photo. Hydatid cyst was perfectly identified (31/31). Among Mecistocirrus digitatus classes, adult was recognized with 25/26 correct predictions, while the anterior and tail portion subclasses showed slight inter-class confusion, particularly between each other (3 misclassified between anterior and tail). Moniezia microscopic photo was identified without any errors (57/57). For Setaria digita adult, 50 out of 52 samples were correctly classified, with two samples misclassified as Toxocara vitulorum adult. For classes with smaller sample sizes, such as Strongyle and Strongyle larvae, the model still performed robustly, achieving 22/23 and 22/24 correct classifications, respectively, with limited cross-confusion between these two morphologically similar categories. Finally, Toxocara vitulorum adult achieved 42 correct classifications out of 47, with minor overlap with Fasciola gigantica and Setaria digita adult. Toxocara vitulorum egg achieved 14 correct out of 15, with one instance misclassified as Hydatid cyst.

To assess the stability and reliability of the proposed CNN model, it was trained *N* = 10 times under identical hyperparameter settings, with different random seeds for each run shown in Table 4. The results, summarized in Table 5, demonstrate consistent performance across runs. The Macro F1-score averaged 0.9527 ± 0.0021 (mean ± standard deviation), with a 95% confidence interval (CI) of [0.9513, 0.9540], while the Weighted F1-score averaged 0.9598 ± 0.0019 (95% CI: [0.9587, 0.9610]). The overall accuracy achieved 0.9616 ± 0.0024 (95% CI: [0.9601, 0.9630]). These narrow confidence intervals and low standard deviations confirm that the model’s performance remains stable and robust across multiple independent training runs, indicating strong generalization capability and low sensitivity to initialization variations.

**Table 4:** Accuracy, macro F1 and weighted F1 score for multiple Execution.

Run	Accuracy	Macro F1	Weighted F1
1	0.962	0.953	0.9603
2	0.96	0.9511	0.9603
3	0.963	0.9534	0.9621
4	0.965	0.9561	0.9567
5	0.959	0.9508	0.9572
6	0.96	0.9508	0.9601
7	0.965	0.9562	0.959
8	0.963	0.9538	0.9623
9	0.958	0.9501	0.9592
10	0.961	0.9517	0.9617

**Table 5:** Accuracy and F1-Score performance with confidence Intervals.

Metric	Mean	Standard deviation	95% CI (lower—upper)
Accuracy	0.9616	0.0024	0.9601–0.9630
Macro F1	0.9527	0.0021	0.9513—0.9540
Weighted F1	0.9598	0.0019	0.9587—0.9610

Although pre-trained architectures such as DenseNet, MobileNet, and ResNet50 achieve slightly higher overall accuracy (98.64% for DenseNet and MobileNet, 84.63% for ResNet50), our Custom CNN demonstrates clear advantages for parasite detection. As shown in Table 6, the Custom CNN achieves a Macro F1-score of 0.9517 and a Weighted F1-score of 0.9617, reflecting balanced performance across all 16 classes, whereas DenseNet and MobileNet, despite higher overall accuracy, show slightly less consistency for underrepresented parasite classes, and ResNet50 performs poorly on this specialized dataset due to its general-purpose training. Designed with only four convolutional layers (32, 64, 128, 256 filters), our network is computationally efficient, enabling faster training and inference compared to deeper models like DenseNet and ResNet50, which require substantially more resources. Moreover, while MobileNet is lightweight, its feature extraction is less tailored to the specific morphological patterns of parasites, resulting in slightly lower class-wise robustness. In contrast, our Custom CNN is specifically optimized for the parasite dataset, capturing relevant morphological features with high fidelity, making it robust, accurate, and deployable on resource-constrained devices, which is critical for practical veterinary applications.

**Table 6:** Performance comparison of custom CNN with established architectures.

	Custom_CNN	DenseNet	MobileNet	ResNet50
Accuracy	0.9616	0.9864	0.9864	0.8463
Macro Avg	0.9517	0.9807	0.9809	0.8949
Weighted Avg	0.9617	0.9865	0.9866	0.9098

**Fig. 5:**
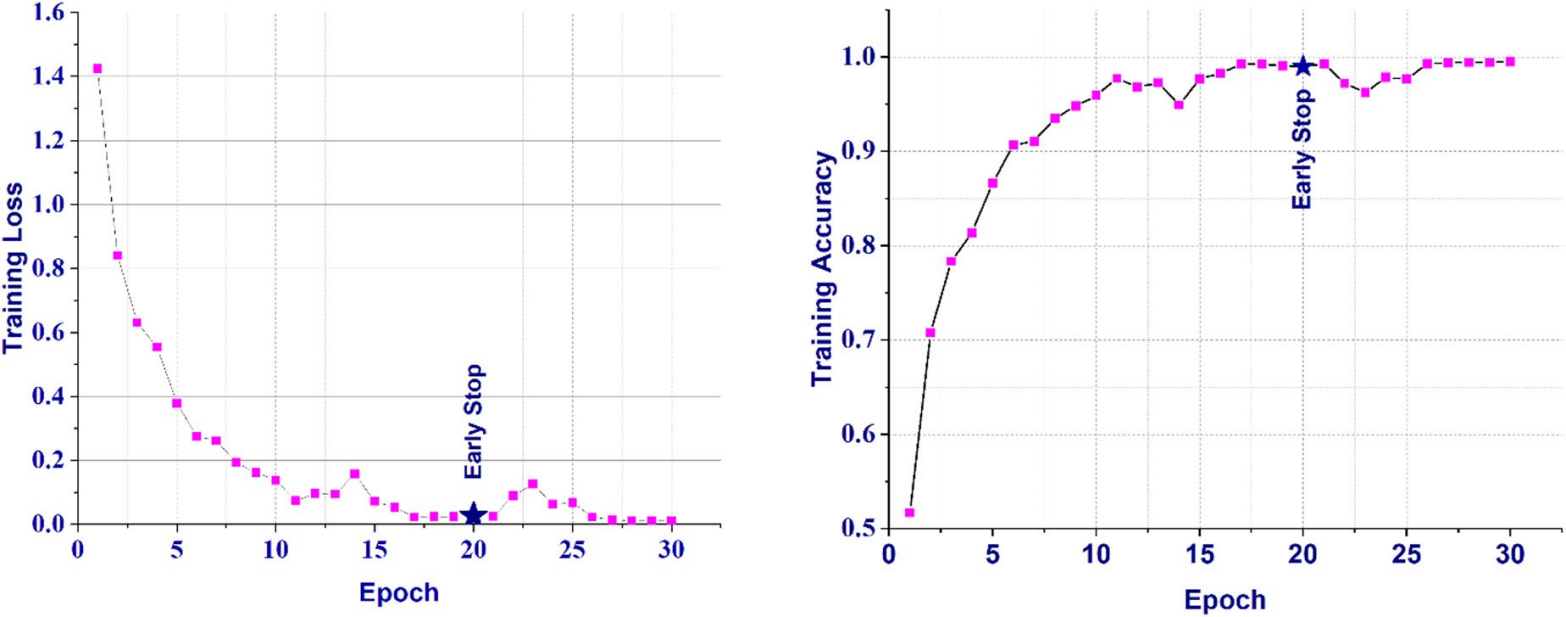
Training Loss and Accuracy Curve for the proposed model.

**Fig. 6:**
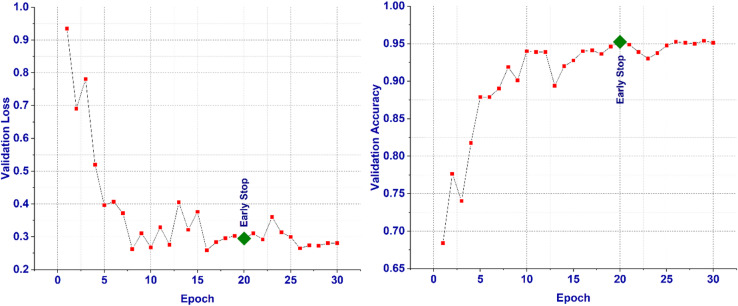
Validation Loss and Accuracy Curve for the proposed model.

Figures 5 and 6 illustrates the training and validation loss and accuracy curves over 30 epochs. The validation accuracy reached its peak at epoch 20 (95.26%), corresponding to the early stopping point. Beyond epoch 22, the validation loss began to fluctuate while the training accuracy continued to improve, indicating the onset of mild overfitting. The consistent gap between training and validation accuracy remained minimal until epoch 20, suggesting strong generalization capability of the proposed CNN model.

The system is further equipped with a PHP-based web interface, allowing seamless integration and real-time online predictions via an API utilizing the trained .h5 model. This interface ensures a user-friendly experience and accessibility for end-users. The model is scalable and compatible with low-resource hardware, making it deployable on web or cloud-based platforms. Furthermore, its adaptability enables easy integration with other applications, allowing for versatile deployment across various operational environments while maintaining high accuracy and efficiency. The developed model has been submitted for copyright protection under Intellectual Property India, Govt of India (Diary Number: 34207/2024-CO/SW). The utility of model also applied under Intellectual property protection in the form of Patent, Govt of India (Application number 202531006472 A; on dated 31.01.2025).

## Discussion

AI technology is transforming multiple facets of animal science through innovative technologies and methodologies^7,8^. Recent innovations include convolutional neural networks (CNNs) for giant panda identification^9^, predictive models for dairy production^10^ and accuracy in breeding programs^11^. AI-driven systems are also used for detecting farrowing activity in sows^12^ poultry management through intelligent robotics^13^ and smart duck counting^14^. Nutritional management has advanced through intelligent feeding systems for livestock^15^ while automated health monitoring has evolved with predictive models for various physiological parameters^16,17^ and comprehensive tracking systems^18,19^. Diagnostic capabilities now include body temperature monitoring and prediction^20^, and automated welfare assessment systems^21^, as well as computational models enabling risk prediction^22^, preventive interventions and identifying subtle patterns in animal behaviour indicating early disease onset^23–25^.

These technological innovations deliver substantial benefits to animal welfare and agricultural productivity, particularly in resource-constrained environments like the Northeast Hill (NEH) region of India, where parasitic infections significantly impact economic outcomes and animal health. The integration of AI-powered diagnostic tools into local veterinary practice offers more rapid, consistent, and accurate disease identification compared to traditional methods, particularly in areas with limited access to specialized expertise.

The application of AI for parasite detection has demonstrated remarkable progress across diverse contexts. CNNs have effectively quantified tick burdens on cattle using infrared thermographic imaging, with models achieving training accuracies of 90–95% for both engorged and unfed ticks^26^. This approach addresses tick-borne diseases threatening approximately 80% of the global cattle population. CNN-based systems for rapid cattle disease diagnosis have achieved 95% accuracy in classifying visible symptoms captured via mobile devices^27^, while deep learning models have successfully detected lumpy skin disease in cattle using transfer learning^28^. Beyond livestock applications, CNNs have identified snails and parasites relevant to human schistosomiasis^29^, and fully convolutional networks have enabled pixel-level segmentation in parasite detection from microscopic cattle samples^30^. Other researchers have developed deep neural network-based image retrieval systems for detecting anaplasmosis in cattle using the Resnext-50 model^31^ and demonstrated the importance of data augmentation techniques in cattle segmentation, achieving 99.5% mean accuracy and 97.3% mean IoU in complex backgrounds^32^.

Building on these advancements, our AI-powered computer vision system employs sophisticated feature extraction methods to transform microscopic images into reliable diagnostic tools. The field of microscopic parasite identification presents unique challenges compared to macroscopic symptom recognition or external parasite detection, requiring architectures capable of distinguishing subtle morphological features across diverse taxonomic groups. While several approaches have been explored in the literature, including complex architectures such as Resnext-50^31^, our findings suggest that carefully optimized conventional CNN architectures can achieve comparable performance for specialized classification tasks.

Our CNN architecture, with its four convolutional layers using progressively increasing filters (32, 64, 128, 256), enables hierarchical feature extraction that achieved 96% accuracy in just 30 epochs when classifying 16 different parasite eggs and adults. The integration of our model with a user-friendly PHP-based application allows veterinarians to upload microscopic images for instant parasite identification, demonstrating practical implementation of AI in field conditions. This approach significantly reduces human error, enables early intervention, and supports veterinary practices in resource-constrained environments. Recent literature has increasingly emphasized the importance of such practical deployment considerations alongside theoretical advancements in model architecture^26,27^. Our system addresses this need through a web-based interface accessible via commonly available devices, minimizing barriers to adoption in rural veterinary settings.

Our system fills a critical gap for the NEH region of India, where parasitic infections constitute a significant threat to livestock health and productivity^33^. The regional focus of our training dataset ensures robustness against the specific parasite populations encountered in this geographical context, addressing a limitation observed in more generalized approaches. Data augmentation and preprocessing techniques, which have shown promise in related fields^32^, were adapted specifically for microscopic imagery to enhance model performance without requiring extensive computational resources during inference.

## Conclusion and future work

This study presents a deep learning–based framework for automated parasite identification using Convolutional Neural Networks (CNNs). The proposed model was trained to classify images of 16 parasite species, encompassing both eggs and adult forms, demonstrating the potential of computer vision to assist parasitological diagnostics and reduce dependence on manual identification. Experimental results show that the model achieved a classification accuracy of 96.16%, with a Macro F1-score of 0.9527 ± 0.0021 and a Weighted F1-score of 0.9598 ± 0.0019 across ten independent runs, confirming the stability and robustness of the approach. These results establish the proposed CNN as an effective and reliable tool for parasite image classification, capable of supporting large-scale and field-level monitoring of parasitic infections, particularly in the livestock sector of the North Eastern region. For future work, expanding the dataset with a wider range of parasite species and life stages, as well as integrating synthetic image generation techniques such as Generative Adversarial Networks (GANs) or Diffusion Models, can further enhance generalization. Model optimization for resource-constrained edge devices would enable real-time, in-field diagnosis, while the inclusion of user feedback mechanisms could support continuous self-improvement of the model. Collectively, these directions will help transition the system from a research prototype to a deployable diagnostic aid, contributing meaningfully to computational parasitology and animal health management.

## Data Availability

The data that support the findings of this study are available from the corresponding author.
